# Scandium effect on the luminescence of Er-Sc silicates prepared from multi-nanolayer films

**DOI:** 10.1186/1556-276X-9-356

**Published:** 2014-07-15

**Authors:** Adel Najar, Hiroo Omi, Takehiko Tawara

**Affiliations:** 1 NTT Basic Research Laboratories, NTT Corporation, 3-1, Morinosato-Wakamiya, Atsugi, Kanagawa 243-0198, Japan; 2 NTT Nanophotonics Center, NTT Corporation, 3-1, Morinosato-Wakamiya, Atsugi, Kanagawa 243-0198, Japan

**Keywords:** Rare-earth doping, Photoluminescence, Thin films

## Abstract

Polycrystalline Er-Sc silicates (Er_*x*_Sc_2-*x*_Si_2_O_7_ and Er_*x*_Sc_2-*x*_SiO_5_) were fabricated using multilayer nanostructured films of Er_2_O_3_/SiO_2_/Sc_2_O_3_ deposited on SiO_2_/Si substrates by RF sputtering and thermal annealing at high temperature. The films were characterized by synchrotron radiation grazing incidence X-ray diffraction, cross-sectional transmission electron microscopy, energy-dispersive X-ray spectroscopy, and micro-photoluminescence measurements. The Er-Sc silicate phase Er_*x*_Sc_2-*x*_Si_2_O_7_ is the dominant film, and Er and Sc are homogeneously distributed after thermal treatment because of the excess of oxygen from SiO_2_ interlayers. The Er concentration of 6.7 × 10^21^ atoms/cm^3^ was achieved due to the presence of Sc that dilutes the Er concentration and generates concentration quenching. During silicate formation, the erbium diffusion coefficient in the silicate phase is estimated to be 1 × 10^-15^ cm^2^/s at 1,250°C. The dominant Er_*x*_Sc_2 - *x*_Si_2_O_7_ layer shows a room-temperature photoluminescence peak at 1,537 nm with the full width at half maximum (FWHM) of 1.6 nm. The peak emission shift compared to that of the Y-Er silicate (where Y and Er have almost the same ionic radii) and the narrow FWHM are due to the small ionic radii of Sc^3+^ which enhance the crystal field strength affecting the optical properties of Er^3+^ ions located at the well-defined lattice sites of the Sc silicate. The Er-Sc silicate with narrow FWHM opens a promising way to prepare photonic crystal light-emitting devices.

## Background

The realization of Si photonics requires a series of components, including continuous-wave (CW) coherent light sources, modulators, amplifiers, switches, detectors, and couplers. Great efforts have been made to fabricate these various components, and successes have been achieved to some degree: Modulators based on the electro-absorption effect [1-4] have been demonstrated, Si-based avalanche photodetectors with a 340-GHz gain bandwidth product have been realized [5], a nanophotonic switch has been made by IBM [6], and on-chip and off-chip couplers have also been demonstrated [7,8]. Among these components, coherent light sources and amplifiers are the most challenging because of the lack of a Si-compatible high-gain material. Bulk Si is a very inefficient emitter because of its indirect bandgap. An alternative approach is to introduce rare-earth ions as impurities into Si [9]. Erbium-doped materials are widely studied as active media in planar Si-compatible optical amplifiers [10,11] owing to the radiative emission of erbium at 1.54 μm, which is a strategic wavelength for telecommunications [12-14].

However, obtaining room-temperature erbium luminescence from doped silicon is not easy, mainly because of the low solubility of erbium in bulk silicon, small emission cross section, and very strong non-radiative coupling between erbium ions and the silicon host. The time constant of the non-radiative transfer mechanisms is much shorter than that of the erbium luminescence (microseconds and milliseconds, respectively) [13,15]. A promising solution could be the use of rare-earth (RE) compounds, which permit us to gradually insert Er ions inside a proper crystalline structure, by substituting RE ions with Er ions, and thus avoid their clusterization [16]. Recently, Er silicates have been reported by many researchers as a possible alternative [17,18] to demonstrate optical amplification. Er, a major constituent instead of a dopant, can provide optically active Er concentrations that exceed 10^22^ cm^-3^ [19]. However, pure Er silicates are not suitable for 1.54-μm applications as the extremely high Er concentration leads to effects such as concentration quenching and cooperative up-conversion, which introduce strong non-radiative recombination paths for the 1.54 μm luminescence [19,20]. Lo Savio et al. have shown that Y-Er disilicate (Y_2-*x*_Er_*x*_Si_2_O_7_) is a good host candidate since it affords a maximum solubility of 10^22^ cm^-3^, which is due to the same crystalline structure with very similar lattice parameters in the constituent materials (Er_2_Si_2_O_7_ and Y_2_Si_2_O_7_) and because both Er and Y atoms occupy the same atomic sites [21]. Scandium ions (Sc^3+^), on the other hand, present a smaller size (ionic radius = 0.75 Å) compared to erbium (Er^3+^) (ionic radius = 0.881 Å). Generally, this can result in enhancing crystal field strength for Er dopants, silicates, and oxides [16,22]. In fact, Fornasiero et al. synthesized single crystal of Er-doped Sc silicates using the Czochralski technique with the idea that Sc^3+^ ions can increase the Stark splitting of the thermally populated erbium ground state as well as of other electronic energy levels of the silicates and therefore reduce reabsorption losses [16]. However, thin film growth of Er-Sc silicates on silicon wafer has not been established, and thus, the optical properties of the silicate have not been sufficiently characterized yet, compared with those of Er-Y silicates.

In this work, we have synthesized a polycrystalline Er-Sc silicate compound (Er_*x*_Sc_2-*x*_Si_2_O_7_) in which Er and Sc are homogeneously distributed using RF sputtering with multilayer Er_2_O_3_, Sc_2_O_3_, and SiO_2_ layers deposited on SiO_2_/Si (100) substrate and thermal annealing at high temperature. The diffusion coefficient of Er was determined after annealing at 1,250°C. The photoluminescence of the dominant phases of the Er-Sc silicate was reported and discussed.

## Methods

Er-Sc multilayer thin films were grown by RF sputtering by alternating 15-nm-thick layers of Er_2_O_3_ and Sc_2_O_3_ separated by a 15-nm-thick SiO_2_ layer. These layers were deposited on 50-nm-thick Er_2_O_3_ on SiO_2_ (1.3 μm)/Si (100) substrate at room temperature. After deposition, the samples were annealed in O_2_ at 1,250°C for 1 h. The samples were analyzed by atomic force microscopy (AFM), using cross-sectional transmission electron microscopy (TEM)/energy-dispersive X-ray spectroscopy (EDS) images obtained at 200 keV, and by synchrotron radiation grazing incidence X-ray diffraction (GIXD) experiments that were performed on the as-grown and annealed samples at the BL24XU in SPring-8 (Hyogo, Japan) using an X-ray wavelength of 1.24 Å and an incidence angle of 1.0° [23]. Photoluminescence (PL) measurements were performed using a laser at 1,527.6 nm with an excitation power of 125 mW at 4 and 300 K. The excitation laser was focused to a spot with a diameter of about 15 μm and an incident angle of 45° through an objective lens. The luminescence from the sample was collected perpendicularly with a different objective lens with a numerical aperture of 0.40 [24]. The PL spectra were detected using a 0.5-m spectrometer and cooled InGaAs detector [23,25].

## Results and discussion

**Figure 1:**
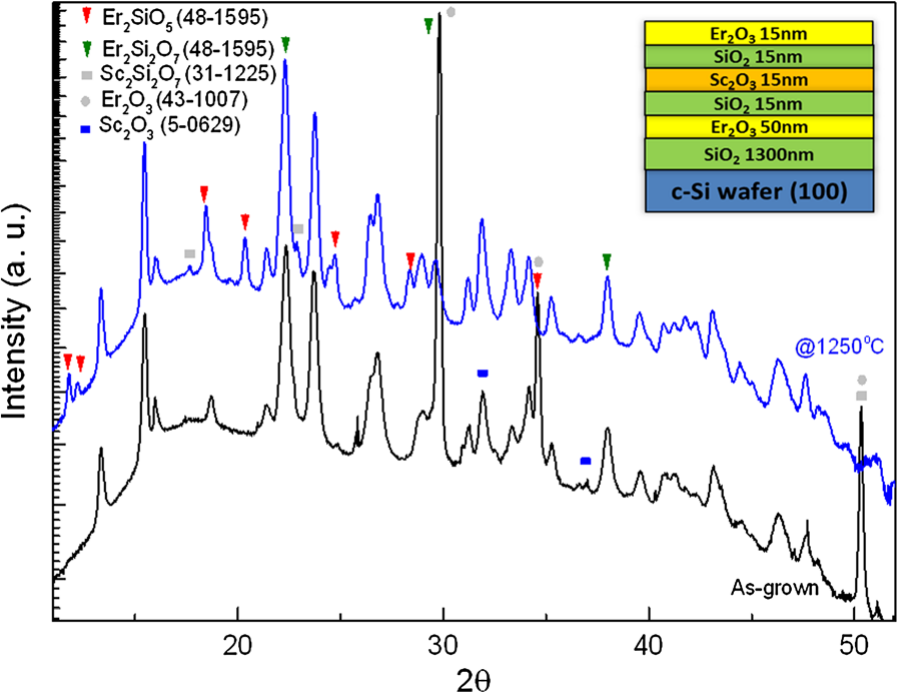
**Synchrotron radiation GIXD obtained from the samples after deposition and annealing at 1,250°C for 1 h in O**_**2**_**.** The Joint Committee on Powder Diffraction Standards (JCPDS) numbers correspond to different compounds. The inset shows the fabricated structure.

GIXD profiles of the crystalline structure after the deposition and annealing of the films are shown in Figure 1. The inset image illustrates the multilayer structure before annealing. The GIXD profile of the sample after deposition shows the presence of Er_2_O_3_, Er_2_Si_2_O_7_, and Sc_2_Si_2_O_7_ in the films. After the annealing at 1,250°C, peaks with high intensity are assigned to Er_2_Si_2_O_7_ and Er_2_SiO_5_ phases. After annealing, we have only Er_2_Si_2_O_7_ and Er_2_SiO_5_ because of the diffusion of Er and Sc in different layers and the formation of new polycrystalline mixed compounds assigned to Er_*x*_Sc_2-*x*_Si_2_O_7_ and Er_*x*_Sc_2-*x*_SiO_5_. Moreover, it has been demonstrated that in the Yb-Er disilicate or Y-Er disilicate, Er^3+^ can be substituted with Y^3+^, Yb^3+^, or Tm^3+^ ions because they have similar ionic radii, whereas Sc^3+^ ions have small radii that affect the crystalline structure of the Er-Sc silicate.

**Figure 2:**
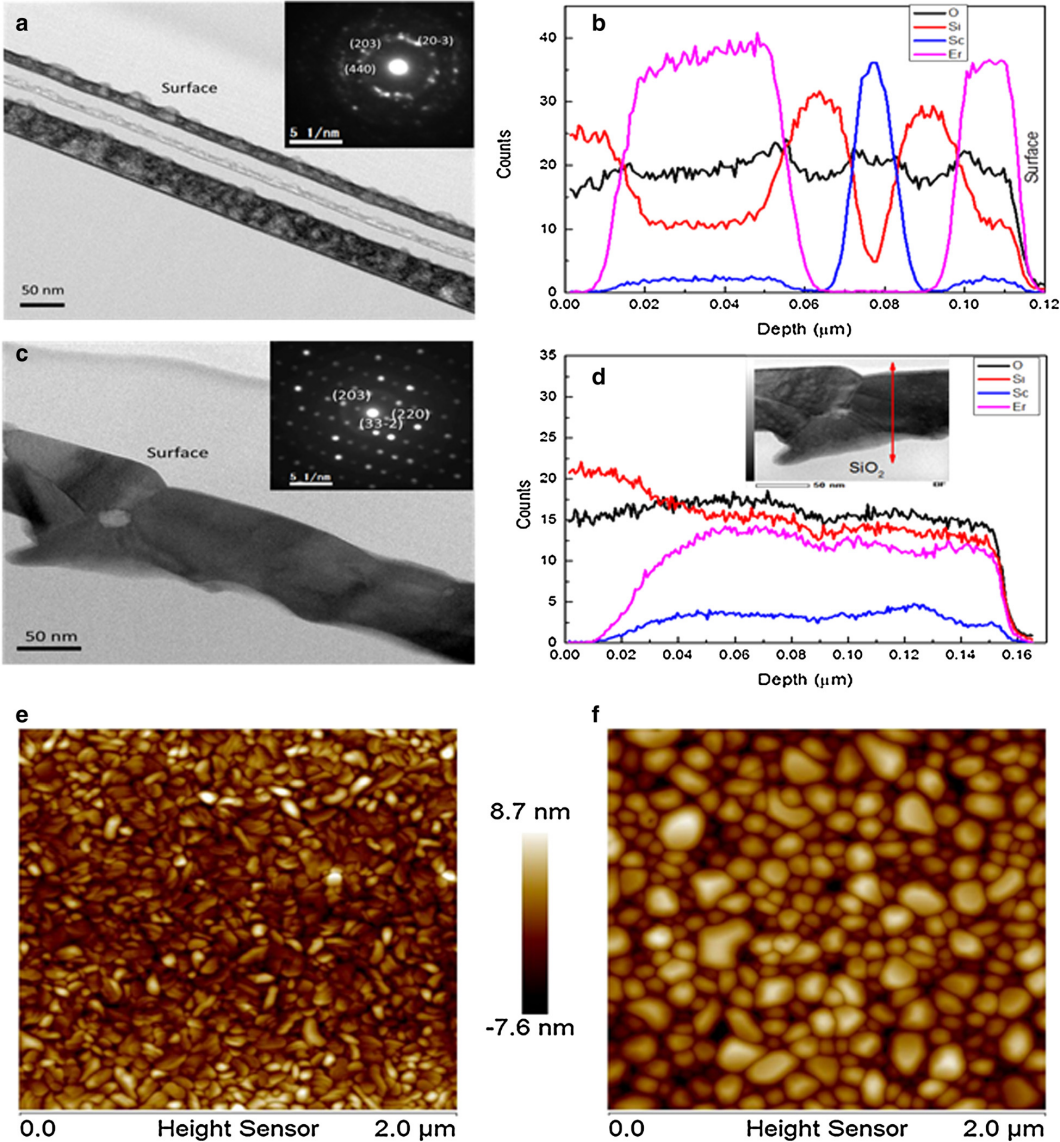
**Cross-sectional TEM images, EDS concentration profiles, and AFM images. (a, c)** Cross-sectional TEM images before and after annealing at 1,250°C with SAED images in the insets. **(b, d)** EDS concentration profiles of Er, Sc, O, and Si for the corresponding inset TEM images **(a)** and **(c)**, respectively. **(e, f)** AFM images of the sample after deposition and annealing at 1,250°C.

To determine the microscopic structures of the existing phases (Er_*x*_Sc_2-*x*_SiO_5_, Er_*x*_Sc_2-*x*_Si_2_O_7_, Er_2_O_3_) after deposition, we performed TEM analysis of the cross section coupled to EDS measurements and selected area electron diffraction (SAED) images of the samples after deposition and annealing at 1,250°C. The cross-sectional image in Figure 2a obtained after deposition shows different layers of Er_2_O_3_, Sc_2_O_3_, and SiO_2_ with a total deposition thickness of around 109 nm. In Figure 2a, the inset SAED image from the Er_2_O_3_ layer at the bottom shows multicrystalline rings. The interplanar spacings (*d*) are about 1.29, 1.32, and 1.52 Å, corresponding respectively to (203), (440), and (20-3) planes, for Er_2_Si_2_O_7_ and 1.32 and 1.52 Å, corresponding respectively to (800) and (444) planes, for Er_2_O_3_. The same phases (Er_2_Si_2_O_7_ and Er_2_O_3_) are identified in the top layer of Er_2_O_3_. The corresponding EDS profiles obtained from the samples after deposition are shown in Figure 2b with discontinuity of Er and Sc concentration profiles in the deposition layers.

After thermal treatment at 1,250°C in O_2_, we formed a unique layer with an average thickness of 102 nm as shown in Figure 2c. The SAED images show a single-crystal compound. The interplanar spacings are 1.30, 1.54, and 2.61 Å, corresponding respectively to (203), (33-2), and (220) planes, for Er_2_Si_2_O_7_. The annealing treatment at 1,250°C results in the intermixing between different layers with homogeneous concentration profiles of Er, Sc, Si, and O in depth (Figure 2c). Indeed, Er and Sc diffuse in the SiO_2_ layer. EDS measurements show that Er and Sc concentrations are 6.7 × 10^21^ and 1.4 × 10^21^ atoms/cm^3^, respectively, with the Er/Sc ratio of 4.5. This high concentration of Er incorporated into the Sc_2_O_3_ matrix is due to the presence of Sc that creates concentration quenching. From the GIXD and TEM analysis, we conclude that Er_2_Si_2_O_7_ is in the bottom and top layers before annealing and that the Er_*x*_Sc_2-*x*_Si_2_O_7_ phase is dominant after annealing at 1,250°C. In addition, it is considered that the high-temperature annealing at 1,250°C and long annealing time enhance the reaction of Er-O and Si-O precursors with the SiO_2_ interlayers, converting most of the Er_2_SiO_5_ to Er_2_Si_2_O_7_ [18]. The existence of the Er_*x*_Sc_2-*x*_SiO_5_ phase after annealing determined by GIXD analysis may be due to size of the analyzed surface which is much bigger using an X-ray beam than a TEM electron beam. The surface morphology after deposition and annealing was analyzed by AFM. The AFM images in Figure 2e,f show a flat surface with no cracks after annealing up to 1,250°C. After deposition, the roughness value of approximately 2.7 nm was measured against that of 4.1 nm after annealing because of the increase of the grain size.

**Figure 3:**
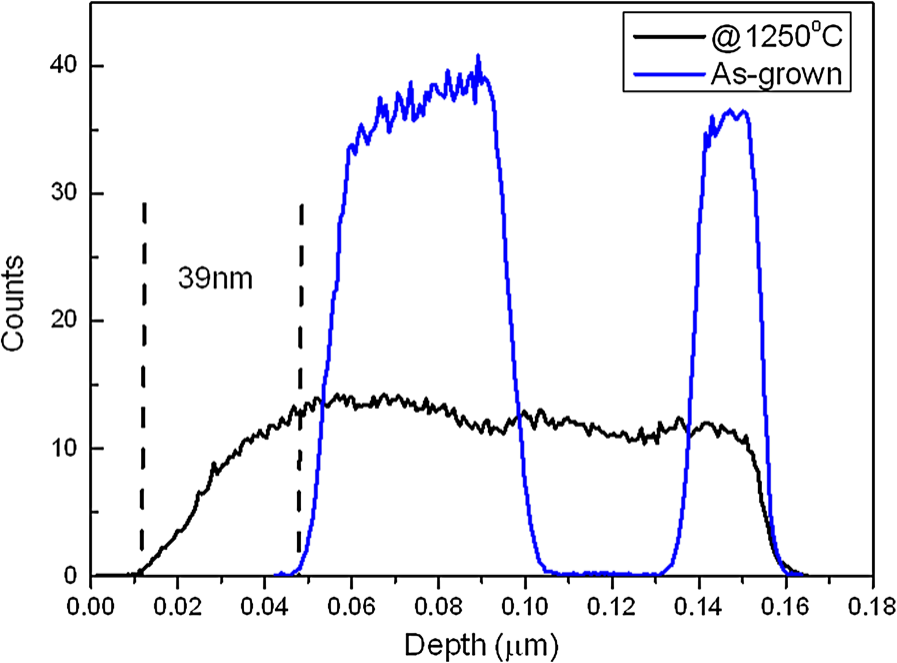
EDS concentration profiles of Er after deposition and annealing at 1,250°C.

Er diffusion at 1,250°C was analyzed by measuring the Er concentration profiles before and after heat treatment in Figure 3. After deposition, the atomic weight of Er is estimated to be 35% to 40%, and these values decrease from 11% to 14% after annealing at 1,250°C due to the homogeneous redistribution of Er atoms in the annealing layers. Er diffuses in the depth with a diffusion length of around 39 nm in the bottom layer of SiO_2_ compared to the as-grown sample (Figure 3), but we suppose that Er diffuses with the same thickness in the other layers. The diffusion length is given by $L=2\sqrt{\mathrm{Dt}}$, where *D* is the diffusion coefficient and *t* is the duration of the thermal treatment. For the annealing temperature of 1,250°C, the diffusion coefficient *D* is 1 × 10^-15^ cm^2^/s. This value is fairly consistent with the value of 0.63 × 10^-15^ cm^2^/s for Er in the SiO_2_ layer prepared by magnetron sputtering and annealed at 1,100°C for 1.5 h in N_2_[26] and about 2 orders of magnitude higher than the diffusion coefficient of silicon-rich silicon oxide (SRSO) of 1.2 × 10^-17^ cm^2^/s at 1,100°C [27].

**Figure 4:**
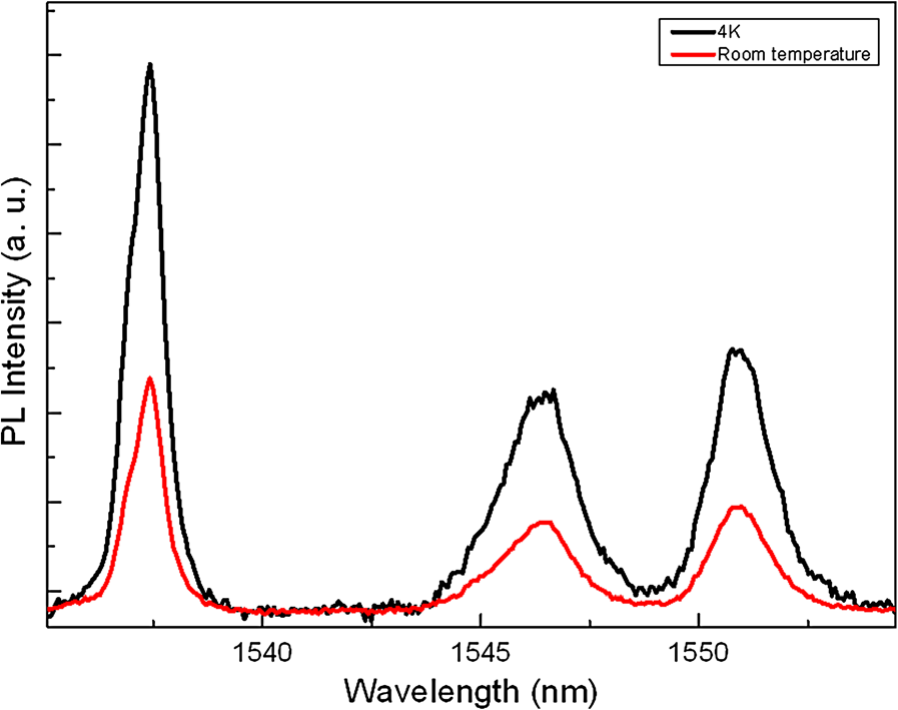
PL spectra at room temperature and 4 K obtained from the sample annealed at 1,250°C.

The PL in the range from 1,533 to 1,555 nm was measured in the sample annealed at 1,250°C, at 4 K, and at room temperature using 1,527.6-nm excitation wavelength, which corresponds to the energy between the ground state (^4^I_15/2_) and second higher excited state (^4^I_13/2_), with 125-mW excitation power. As shown in Figure 4, PL spectra exhibit the same shape for both temperatures with the main emission peak at 1,537 with sub-peaks at 1,546.2 and 1,551 nm corresponding to the energy levels of Er^3+^ ions. The peak at 1,537 nm corresponds to the energy between Er^3+^ (^4^I_15/2_) and Er^3+^ (^4^I_13/2_) ions in the Sc silicate phase with the full width at half maximum (FWHM) of 1.6 nm at room temperature and 4 K. We attribute this enhancement to the narrow emission peak of Er_*x*_Sc_2-*x*_Si_2_O_7_ to the well-defined lattice sites for Er^3+^. This narrow emission will be very promising for photonic crystal light-emitting devices because the extraction efficiency can be increased with a pronounced narrowing of the emission. Shin and Lee have shown a peak emission at 1,529 nm with an FWHM of 11 nm for Er_*x*_Y_2-*x*_SiO_5_ annealed at 1,200°C using an excitation wavelength of 488 nm [28]. In addition, Miritello et al. obtained a peak emission at 1,535 nm for α-(Yb_1-*x*_Er_*x*_)_2_Si_2_O_7_ with a 37-nm FWHM using 532 nm excitation wavelength after annealing at 1,200°C [29]. The GIXD and SAED results confirm the emission peaks corresponding to the dominant Er_*x*_Sc_2-*x*_Si_2_O_7_ phase. Furthermore, the peak energies are different from the Stark level splitting of Er energy levels in Er-doped Sc_2_Si_2_O_7_ and Sc_2_SiO_5_ single crystals at low temperature identified by Fornasiero et al. [16] and Omi et al. [30]. Since both Sc and Y are optically inactive in the matrix, in this way, it is possible to control the Er pair interactions and maximize the Er active concentration. The advantage of using Sc in comparison to Y is that the radius of Sc is smaller compared to those of Y and Er. This smaller radius enhances the crystal field strength which affects the luminescence properties with smaller FWHM compared to the effect of Y. However, Er can be substituted with Y in the silicate phase which is not the case for Sc due to the radius effect.

The crystal field strength parameters are defined by [31] $N_{V}={\left[\sum_{k,q}\frac{4\pi }{2K+1}{\left(B_{q}^{k}\right)}^{2}\right]}^{1/2}$, where $B_{q}^{k}$ is the crystal field parameters that affect the Stark levels of Er^3+^, which characterize the interaction between ligands and the central ions and include the radial integral of the wavefunction. The values of the crystal field strength parameters $N_{V}$ have been calculated for Er^3+^ doped in different matrices: Sc_2_O_3_ (5,300 cm^-1^), Y_2_O_3_ (2,700 cm^-1^), and Er_2_O_3_ (2,200 cm^-1^) [32,33], and show clearly that the crystal field strength increases regularly with decreasing ionic radius of the RE host cation, which generates the Stark splitting energy levels. In our case, the 8-nm redshift is due to the presence of Sc ions, which increase the crystal field strength and thereby enhance the Stark splitting of the thermally populated Er energy levels (^4^I_15/2_ and ^4^I_13/2_ levels) as well as that of the other electronic energy levels.

## Conclusions

In summary, a polycrystalline Er_*x*_Sc_2-*x*_Si_2_O_7_-dominant compound was fabricated using RF sputtering by alternating Er_2_O_3_ and Sc_2_O_3_ layers separated by a SiO_2_ layer and annealed in O_2_ gas. After high-temperature annealing at 1,250°C, the Er and Sc ions are distributed homogeneously in the layer. The erbium diffusion coefficient in the SiO_2_ at the annealing temperature was estimated to be 1 × 10^-15^ cm^2^/s. The Er-Sc silicate layer shows a sharp emission peak at room temperature centered at 1,537 nm as a result of the strong crystal field strength generated by the small ionic radii of Sc^3+^ ions. The Er-Sc silicate could be used as an efficient material for photonic devices.

## Competing interests

The authors declare that they have no competing interests.

## Authors' contributions

AN designed and fabricated the structure and carried out the experiments as well as the analyses. HO carried out the GIXD experiments and the analysis of data. TT carried out the PL measurements and the analysis of data. All authors read and approved the final manuscript.
